# Urinary Albumin as a Marker of Future Blood Pressure and Hypertension in the General Population

**DOI:** 10.1097/MD.0000000000000511

**Published:** 2015-02-13

**Authors:** Hiroyuki Takase, Tomonori Sugiura, Nobuyuki Ohte, Yasuaki Dohi

**Affiliations:** From the Department of Internal Medicine (HT), Enshu Hospital, Hamamatsu; and Department of Cardio-Renal Medicine and Hypertension (TS, NO, YD), Nagoya City University Graduate School of Medical Sciences, Nagoya, Japan.

## Abstract

We investigated whether urinary albumin could predict the development of hypertension and future increases in blood pressure in the normotensive general population.

Normotensive subjects who visited our hospital for a physical checkup (n = 6205, men 61.8%, 53.4 ± 11.4 years old) were enrolled in this study. Urine samples were collected for the measurement of albumin concentration, expressed as the ratio of urinary albumin to creatinine concentrations (UACR [mg/g Cr]). After the baseline examination, subjects were followed up for a median of 1089 days with the endpoint being the development of hypertension.

Urinary albumin was in the normal range (UACR <30 mg/g Cr) in most subjects (97.5%). During the follow-up, hypertension developed in 1184 subjects (19.1%, 69.5 per 1000 person-years), with more men than women affected. The incidence of hypertension was increased across the quartiles of UACR by Kaplan–Meier analysis (log-rank, *P* < 0.0001) and the hazard ratio (lowest quartile [median UACR 1.14 mg/g Cr] as reference) was 1.53 (95% confidence intervals 1.30–1.80) in the highest quartile (median UACR 8.87 mg/g Cr). Multivariate Cox hazard analysis in which UACR was taken as a continuous variable identified UACR as a significant predictor of hypertension (hazard ratio 1.37, 95% CI 1.20–1.56). UACR was also an independent predictor of future increases in systolic blood pressure (*P* < 0.01).

Urinary albumin is an independent predictor of hypertension and increases in blood pressure in the general population even in the normal range below the threshold defined for microalbuminuria.

## INTRODUCTION

Hypertension is an established risk factor of cardiovascular morbidity and mortality.^1–3^ While antihypertensive treatment reduces the risk of cardiovascular disease,^4–6^ it is obviously impossible to identify all hypertensive subjects among the general population and managing all such patients in medical facilities could be difficult given the potentially large number. Furthermore, more than half of cardiovascular events occurred in individuals with mild hypertension or normal blood pressure,^3,7–9^ despite the risk reducing with decreasing blood pressure levels. In this context, primary prevention of hypertension is an important public health aim. An intensive targeted strategy focused on identified individuals at highest risk of developing hypertension is an attractive approach for primary prevention of hypertension.

While not the only target organ of high blood pressure, the kidney plays a central role in regulating blood pressure.^10–13^ We previously reported that glomerular filtration rate is a novel predictor of the onset of hypertension and future increases in blood pressure in the general population.^14^ In this earlier study, we also identified proteinuria determined by a dipstick method as a possible predictor of the onset of hypertension, although a definite conclusion could not be drawn because the urinary protein was not quantified.^14^ Moreover, microalbuminuria was independently associated with cardiovascular risk factors such as hypertension, and cardiovascular morbidity in previous studies.^15,16^ These observations suggested the predictive value of lower levels of urinary albumin, that is microalbuminuria, for hypertension. Indeed, microalbuminuria reflects vascular endothelium dysfunction in individuals with and without diabetes^17,18^ and endothelial dysfunction precedes the development of hypertension.^19^ Higher levels of urinary albumin, even within the normal range, were associated with increased risk of hypertension in the Framingham study,^20,21^ although not in other studies in premenopausal women^22^ or in the Multi-Ethnic Study of Atherosclerosis.^23^ In the light of these controversial results, the present study sought to further investigate whether increased excretion of urinary albumin within the low-grade levels (<300 mg/g creatinine) is associated with increased risk of hypertension and future elevations of blood pressure in the general population.

## METHODS

### Study Design

This was a cohort study of participants who visited our hospital for a yearly physical checkup from July 2008 to June 2013 to assess the impact of urinary albumin on the incidence of hypertension. We undertook the study in accordance with the principles of the Declaration of Helsinki and the Ethics Committee of Enshu Hospital approved the study protocol. All participants gave written informed consent to participate prior to the start of the study and at each study visit.

### Study Subjects and Procedures

**FIGURE 1:**
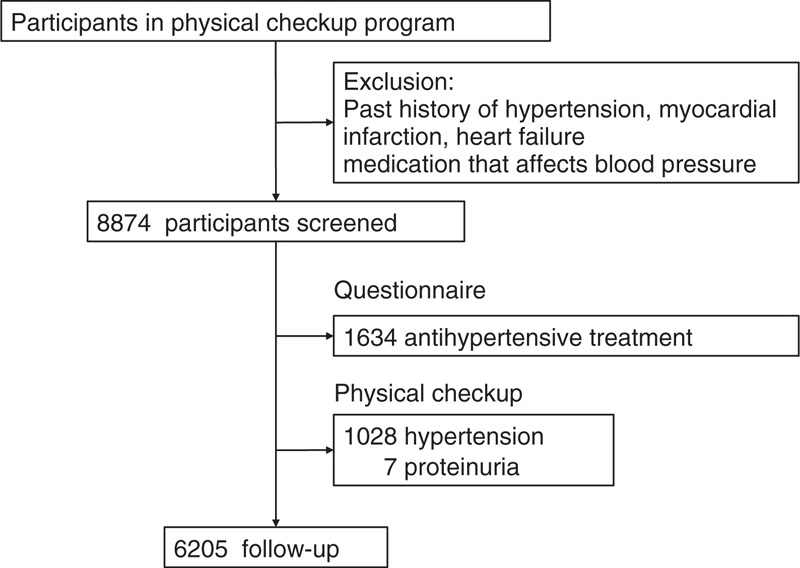
Flow diagram for study participants.

The study subjects were recruited from the group of participants examined between July 2008 and June 2011 (n = 8874). First, individuals under medical treatment for hypertension (n = 1634) were excluded. Among the remaining individuals (n = 7240), the physical checkup (including an interview regarding health status, routine physical examination, chest x-ray, electrocardiography, and laboratory assessment of cardiovascular risk factors) revealed 6212 individuals without hypertension (man 3843, mean age 53.4 years). After further excluding those subjects with proteinuria, 6205 participants (man 3837 [61.8%], mean age 53.4 years, range 22–85 years) were finally enrolled in the present study and followed up with the endpoint being the onset of hypertension (Figure 1). During the follow-up period, blood pressure and urinary albumin were measured once a year at the participant's annual health checkup. The exclusion criteria for the study were a history of hypertension, myocardial infarction, heart failure, or disorders requiring medication that may affect blood pressure. A single-void, morning urine sample was used to measure urinary excretion of albumin. Urinary albumin concentrations were measured by a turbidimetric immunoassay (analytical range ≥1.1 mg/L) and are expressed as the ratio of concentrations of urinary albumin to urinary creatinine (UACR [mg/g Cr]). UACR was recorded as 0 mg/g Cr in subjects with a urinary albumin concentration below the analytical limit. Proteinuria was defined as UACR ≥300 mg/g Cr. Microalbuminuria was defined according to the recommendation of the American Diabetes Association and the National Kidney Foundation (300 > UACR ≥ 30 mg/g Cr).^24,25^

Linear regression analysis was performed for each participant using a change in blood pressure or UACR as a dependent variable and follow-up period (years) as an independent variable, with the slope of the regression line considered as the yearly increase in blood pressure or UACR, respectively. In participants who started antihypertensive medication during the follow-up, changes in blood pressure or UACR were calculated using data obtained before the prescription of antihypertensive drugs. The impact of baseline UACR, changes in UACR during the follow-up period, and longitudinal changes in blood pressure during the follow-up on the onset of hypertension was investigated. Blood pressure was measured using a standard mercury sphygmomanometer with the subject in the sitting position. Three consecutive blood pressure measurements were taken at 2-minute intervals, and the mean of the second and third measurements was recorded as the blood pressure. Subjects were defined as having hypertension with a systolic blood pressure ≥140 mm Hg, diastolic blood pressure ≥90 mm Hg, or if they were taking antihypertensive medications.^3^ Diagnosis of new hypertension was based on blood pressure measured at the participant's annual physical checkup or information obtained from a questionnaire regarding antihypertensive medications. Subjects with high-density lipoprotein (HDL) cholesterol levels <40 mg/dL, low-density lipoprotein (LDL) cholesterol ≥140 mg/dL, triglycerides ≥ 150 mg/dL, or using antidyslipidemic medications were defined as having dyslipidemia.^26^ Subjects with a fasting plasma glucose ≥126 mg/dL or using antidiabetic medications were defined as having diabetes mellitus. The estimated glomerular filtration rate (eGFR) was calculated using a modified formula from the Modification of Diet in Renal Disease study for the Japanese population.^27^

### Statistical Analysis

All analyses were performed using StatView 5.0 (SAS Institute, Inc, Cary, NC). Data presented in the text and tables are expressed as mean ± standard deviation except for serum creatinine, UACR, and follow-up period, which are expressed as the median value and interquartile range. In addition, because the distribution of serum creatinine and UACR was skewed rightward, log-transformed creatinine and (UACR + 0.5) were used for statistical analysis. Differences between 2 means that had a normal distribution were compared by unpaired Student *t* test, and the Mann–Whitney U-test was used to test significance of difference in follow-up periods. Yates’ corrected chi-square (χ2) test was used for comparisons between categorical data. To analyze the endpoint throughout the observation period, the significance of differences in Kaplan–Meier curves was evaluated by the log-rank test and adjusted using multivariate Cox proportional hazard regression models. Hazard ratios and 95% confidence intervals (CIs) were calculated. Furthermore, multivariate Cox proportional hazard regression models were applied to examine the relationship between UACR as a continuous variable and the onset of hypertension. In other series of analyses, multivariate linear regression analysis was performed to assess the relationships of baseline or changes in UACR as well as other baseline variables obtained from the physical checkup program to yearly changes in blood pressure. *P* < 0.05 was considered significant.

## RESULTS

Table 1 lists the baseline characteristics of all participants. The actual follow-up period of the present study was 17,025 person-years and the median follow-up period was 1089 (range 170–1818) days. During the follow-up period, hypertension developed in 1184 subjects (19.1%; 69.5 per 1000 person-years) with a higher incidence in men (22.6%; 82.2 per 1000 person-years) than in women (13.3%; 48.9 per 1000 person-years). Table 2 describes the results of our retrospective analyses, showing characteristics of participants with and without future development of hypertension. Participants who developed hypertension had higher levels of UACR at baseline than those who did not develop hypertension (Table 2). A yearly increase in UACR was higher in participants with than without future hypertension (Table 2).

**TABLE 1:**
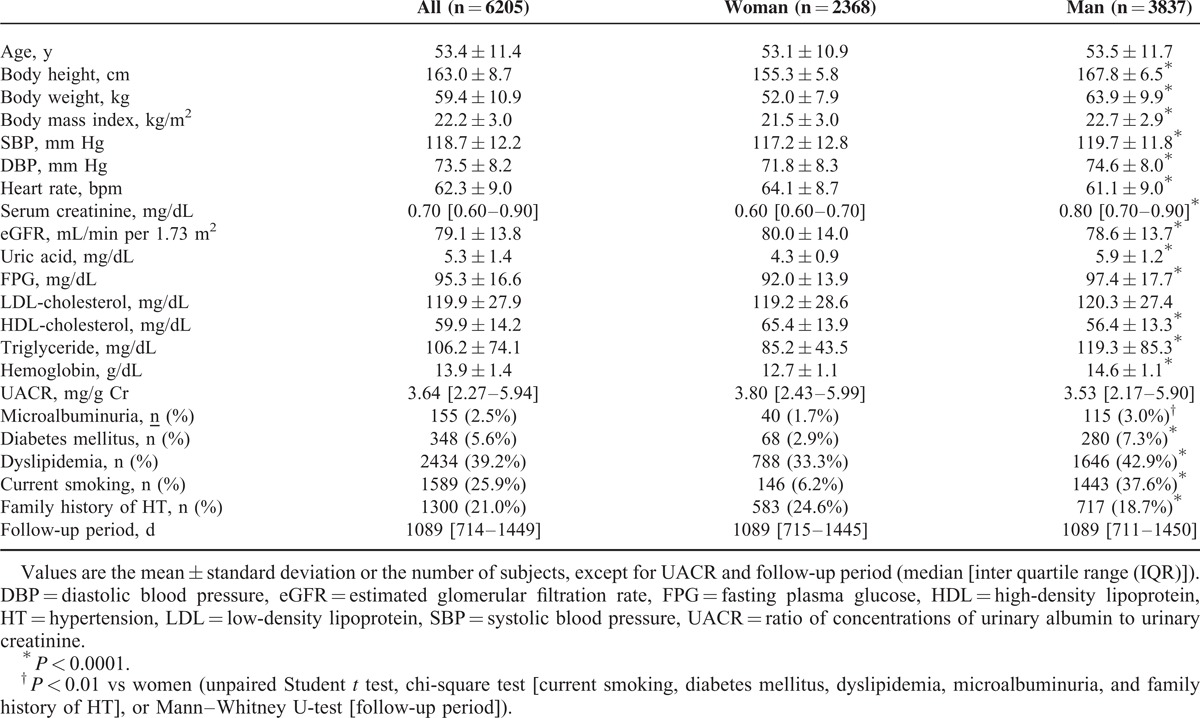
Baseline Characteristics of the Study Subjects

**TABLE 2:**
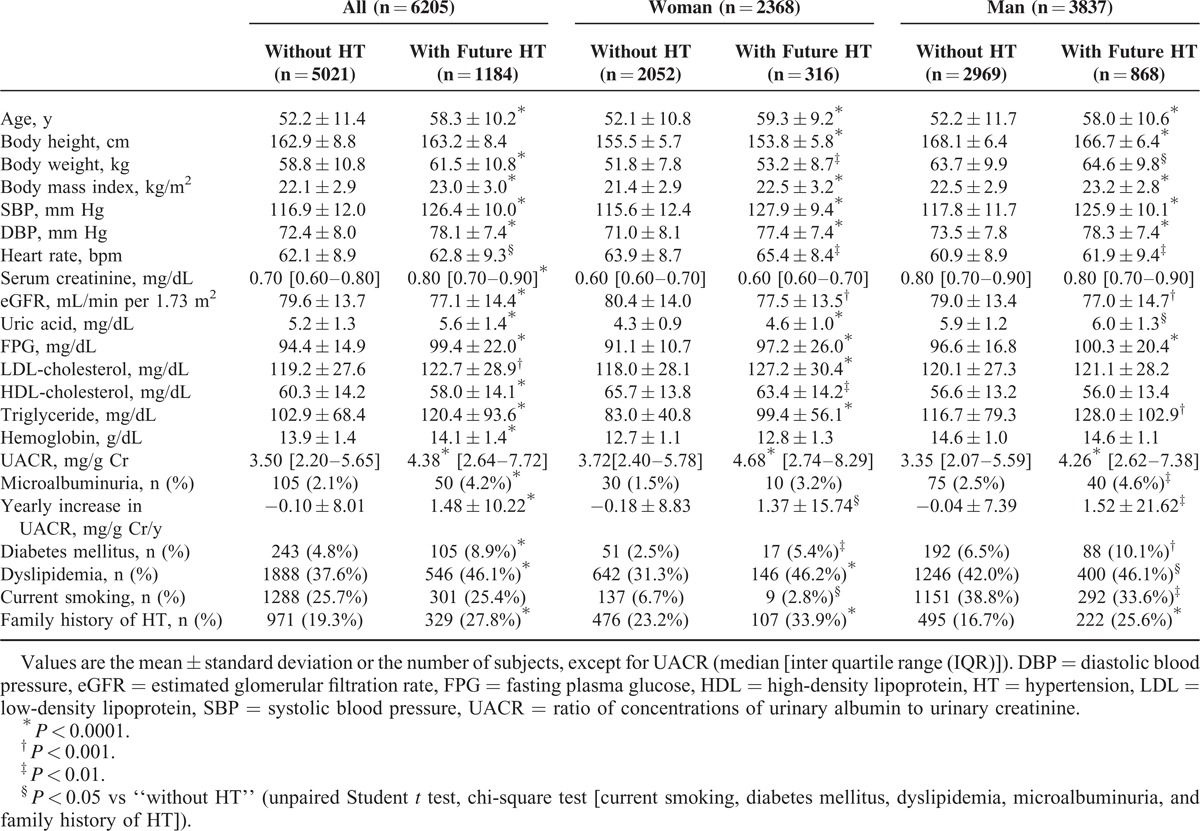
Retrospective Analysis of Study Subjects’ Characteristics at Baseline

**FIGURE 2:**
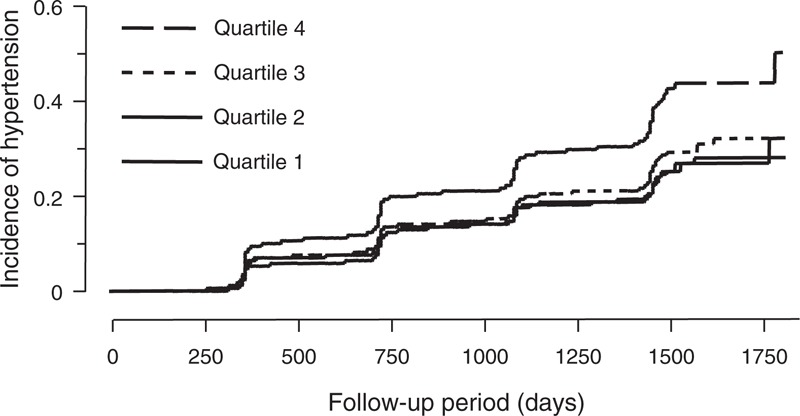
Kaplan–Meier analysis for new onset of hypertension. Participants were divided into quartiles according to their ratio of urinary albumin to urinary creatinine (UACR) concentrations at baseline. The median value of UACR [interquartile range] was 1.14 [0–1.82], 2.97 [2.63–3.28], 4.59 [4.06–5.20], and 8.87 [7.04–14.42] mg/g Cr in the first, second, third, and fourth quartiles, respectively. *P* < 0.0001 by log-rank test.

To evaluate the impact of the UACR on the incidence of hypertension, subjects were divided into 4 groups using the quartiles of UACR at baseline. Kaplan–Meier curve analysis demonstrated that the incidence of hypertension increased across the quartiles of UACR values (56.1, 56.9, 65.4, and 100.3 per 1000 person-years in the first, second, third, and fourth quartiles, respectively, *P* < 0.0001 by log-rank test; Figure 2). The hazard ratio of incident hypertension (first quartile as reference) was 1.090 (95% confidence interval CI; 0.910–1.304), 1.218 (1.023–1.449), and 1.531 (1.300–1.804) in the second, third, and fourth quartiles, respectively, after adjustment for body mass index, systolic blood pressure, heart rate, eGFR, uric acid, fasting plasma glucose, LDL-cholesterol, triglycerides, current smoking habit, and family history of hypertension. Table 3 details the analyses where UACR at baseline was taken as a continuous variable. Unadjusted univariable Cox hazard regression analysis indicated a significant correlation between UACR at baseline and the future incidence of hypertension. In multivariable Cox hazard analysis, UACR was also a significant predictor of incident hypertension (Table 3). Similar results were obtained using a model where serum creatinine was adopted instead of eGFR as an index of kidney function, and age and gender were included (hazard ratio 1.224; 95% CI 1.077–1.391). Exclusion of subjects taking any medication did not alter the results (data not shown). Furthermore, similar results were obtained in a subanalysis of subjects without diabetes mellitus (n = 5857) (hazard ratio 1.678, 95% CI 1.450–1.943, *P* < 0.0001 by univariate analysis; 1.421, 1.235–1.635, *P* < 0.0001, respectively, by multivariate analysis).

**TABLE 3:**
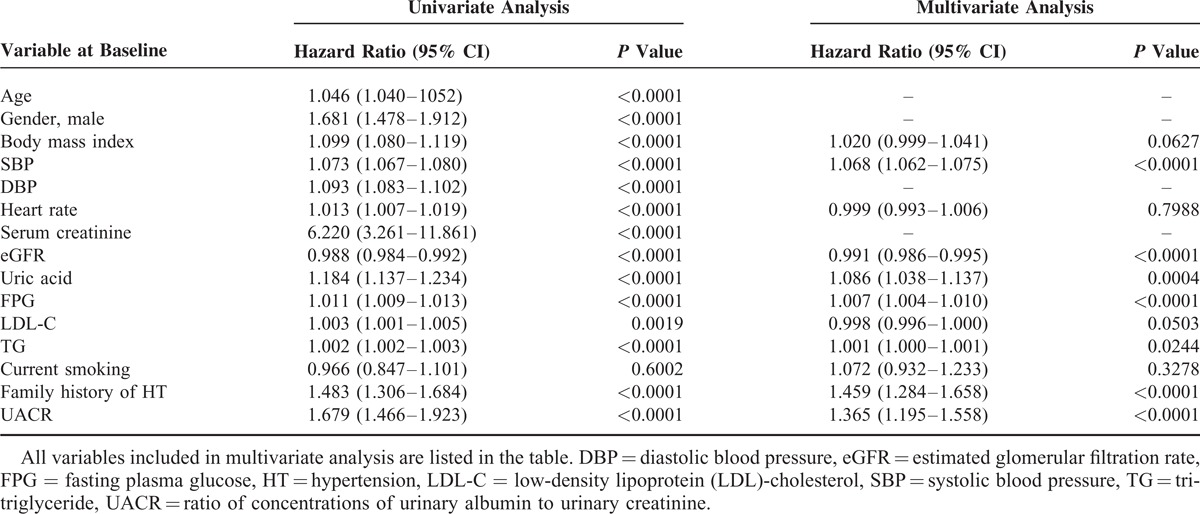
Univariate and Multivariate Cox Proportional Hazard Regression Analyses for Future Development of Hypertension

Other analyses investigated the impact of UACR levels at baseline and yearly changes in UACR during the follow-up period on systolic blood pressure progression. Univariate and multivariate regression analyses indicated that both the baseline levels and any yearly change in UACR positively correlated with a yearly increase in systolic blood pressure (Table 4). Similar results were obtained using a model where serum creatinine was adopted instead of eGFR as an index of kidney function, and age and gender were included (refer to model 3 in Table 4; *r* = 0.032 and *P* = 0.01 for baseline UACR and *r* = 0.044 and *P* < 0.001 for the yearly change in UACR). Exclusion of subjects on any medication did not alter the results (data not shown). In the subgroup of participants without diabetes, baseline UACR, but not the yearly change in UACR, significantly correlated with a yearly increase in systolic blood pressure by both univariate (*r* = 0.037 and *P* = 0.0055, *r* = 0.025 and *P* = 0.064, respectively) and multivariate analyses after adjustment for body mass index, systolic blood pressure, heart rate, eGFR, uric acid, fasting plasma glucose, LDL-cholesterol, triglycerides, current smoking habit, and family history of hypertension (refer to model 3 in Table 4; *r* = 0.055 and *P* < 0.0001 for baseline UACR and *r* = 0.031 and *P* = 0.02 for the yearly change in UACR). Similar results were obtained when a yearly increase in diastolic blood pressure was used as a dependent factor (data not shown).

**TABLE 4:**
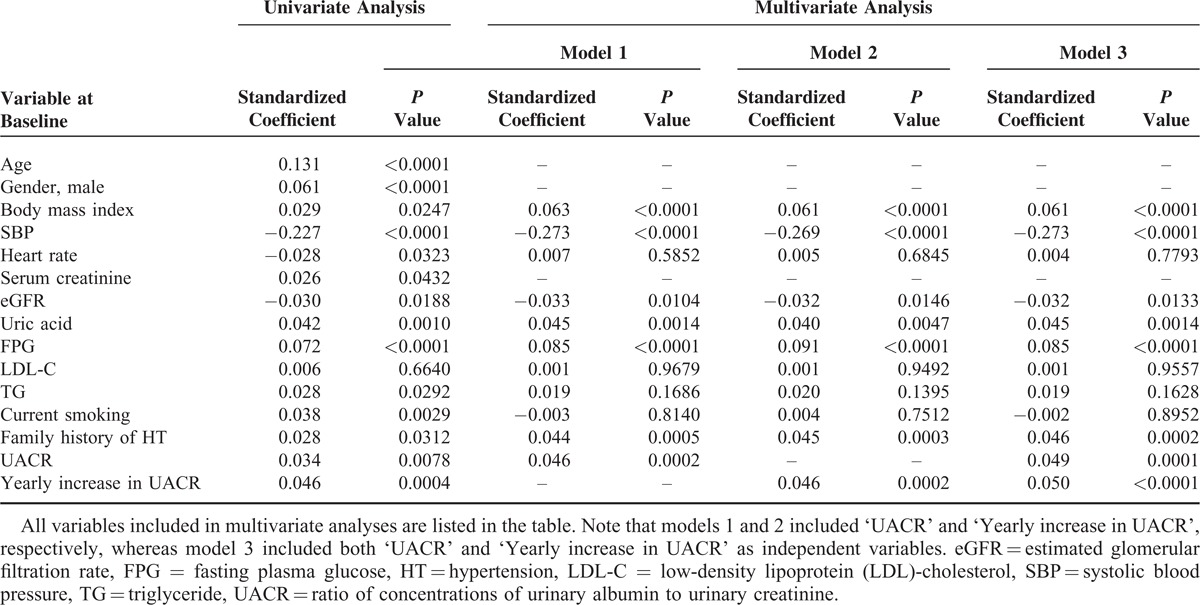
Univariate and Multivariate Regression Analyses Demonstrating the Relationship Between Baseline Variables or the Yearly Change in UACR and the Yearly Increase in SBP

## DISCUSSION

The present study demonstrated that any level of increased urinary albumin was closely associated with the risk for developing hypertension in the Japanese general population. The association between urinary albumin and blood pressure was further confirmed by identifying urinary albumin as a significant predictor of future increases in blood pressure.

Among 6205 subjects without hypertension, Kaplan–Meier curves showed that the risk of developing hypertension increased across the quartiles of UACR, and it was significantly increased in the fourth quartile (median value of 8.87 mg/g Cr) after adjustment for possible factors that could affect the development of hypertension. These findings suggested that excretion of urinary albumin, even levels in the normal range (below the threshold defined for microalbuminuria), should be recognized as an independent predictor for hypertension in the general population, and that the concept of normal range for albuminuria should be reevaluated. The close association between urinary albumin and the development of hypertension was further supported by the finding that UACR taken as a continuous variable was a significant and independent predictor of future hypertension. Although we could not determine whether the risk of hypertension increases in a linear fashion with increasing urinary excretion of albumin or shows a threshold, there could be a threshold of urinary albumin for the development of hypertension considering similar feature of the Kaplan–Meier curves in the first to third quartiles.

The concept that urinary albumin predicts future development of hypertension was further reinforced by the finding that not only UACR at baseline, but also a yearly increase in UACR was independently correlated with the longitudinal increase in systolic blood pressure. The definition of hypertension based on cutoff values is arbitrary due to the continuous and linear relationship between blood pressure and cardiovascular risk.^3,7–9^ Thus, predicting the expected longitudinal increase in blood pressure would be an important clinical aid in understanding individual cardiovascular risk and for identifying suitable persons to undertake intervention programs aimed at primary prevention of hypertension. In this context, the observed close association of urinary albumin with yearly increase in blood pressure has clear clinical and pathophysiological significance.

This observational study does not provide a causal relationship between urinary albumin and the development of hypertension. However, there are several possibilities supporting that urinary excretion of albumin predicts a new onset of hypertension. Most of the albumin filtered through glomeruli is reabsorbed in renal tubules, thus under normal circumstances there should be virtually undetectable urinary excretion of albumin. However, changes in the balance between filtration and reabsorption of albumin in the kidney could contribute, at least in part, to an increase in urinary excretion of albumin, and glomerular endothelial dysfunction could lead to an abnormally increased glomerular filtration of albumin.^28^ Indeed, an inverse correlation was reported between endothelium-dependent relaxations and urinary excretion of albumin.^17,18^ Thus, increased albuminuria might reflect glomerular and/or systemic vascular endothelial dysfunction that precedes hypertension in humans.^19^ In previous cross-sectional studies, urinary albumin was also closely correlated with blood pressure in patients with hypertension^29^ as well as in the general population,^16^ suggesting that the increased level in the present study could be at least partially derived from an elevation of blood pressure. Blood pressure gradually increases during the development of hypertension with significant fluctuations,^30^ indicating that the kidney may be transiently, but frequently, exposed to high blood pressure during these earlier stages of onset. Thus, even a mild increase in urinary albumin could potentially be an early symptom of developing hypertension. Alternatively, glomerular hyperfiltration could underlie an increased urinary excretion of albumin. Reduced nephron number has been implicated as a risk factor for developing hypertension.^31–33^ In individuals with reduced nephron numbers, intraglomerular pressure and glomerular filtration of the residual glomeruli are increased to compensate for the reduced glomerular filtration rate in the kidney, with a consequent increase in urinary excretion of albumin.^34^ Elevated urinary excretion of albumin might therefore in turn be a sign of reduced nephron number.

Several points about this study limited the data interpretation. First, only a single urine specimen was used to assess the baseline urinary excretion of albumin, despite the measurements showing day-to-day variation. Second, data on individual sodium intake were not included in the present analyses, although dietary sodium consumption influences both hypertension and the degree of albuminuria. Third, blood pressure and urinary albumin were measured only once a year during the relatively short follow-up period with a broad range, and new hypertension was ascertained using blood pressure measured at the annual physical checkup or by self-assessment when participants started antihypertensive medications. These points should be kept in mind when interpreting the data.

In conclusion, urinary albumin is a novel predictor of future hypertension and increases in blood pressure in the general population. The risk of developing hypertension increases even with levels of urinary albumin near the threshold defined for microalbuminuria. This finding thus indicates that minor alterations in kidney function could be an important sign for managing blood pressure with the view to preventing hypertension onset.
